# ADAM10 is involved in the oncogenic process and chemo-resistance of triple-negative breast cancer via regulating Notch1 signaling pathway, CD44 and PrPc

**DOI:** 10.1186/s12935-020-01727-5

**Published:** 2021-01-07

**Authors:** Yuanyuan Cheng, Lishuang Lin, Xiaoyan Li, Aiqi Lu, Chenjian Hou, Qian Wu, Xiaomu Hu, Zhongwen Zhou, Zhongqing Chen, Feng Tang

**Affiliations:** 1 Department of Pathology, Huashan Hospital, Fudan University, Shanghai, 200040 China; 2 Department of Surgery, Huashan Hospital, Fudan University, Shanghai, China; 3 Department of Pathology, School of Basic Medical Sciences, Fudan University, Shanghai, China

**Keywords:** A disintegrin and metalloproteinase 10 (ADAM10), Triple-negative breast cancer (TNBC), Notch1 signaling, CD44, Cellular prion protein (PrPc)

## Abstract

**Background:**

Triple-negative breast cancer (TNBC) is the most challenging breast cancer subtype to treat, because it is so aggressive with shorter survival. Chemotherapy remains the standard treatment due to the lack of specific and effective molecular targets. The aim of the present study is to investigate the potential roles of A Disintegrin and Metalloproteinase 10 (ADAM10) on TNBC cells and the effects of combining ADAM10 expression and neoadjuvant chemotherapy treatment (NACT) to improve the overall survival in breast cancer patients.

**Methods:**

Using a series of breast cancer cell lines, we measured the expression of ADAM10 and its substrates by quantitative real-time PCR assay (qRT-PCR) and western blot analysis. Cell migration and invasion, cell proliferation, drug sensitivity assay, cell cycle and apoptosis were conducted in MDA-MB-231 cells cultured with ADAM10 siRNA. The effect of ADAM10 down-regulation by siRNA on its substrates was assessed by western blot analysis. We performed immunohistochemical staining for ADAM10 in clinical breast cancer tissues in 94 patients receiving NACT.

**Results:**

The active form of ADAM10 was highly expressed in TNBC cell lines. Knockdown of ADAM10 in MDA-MB-231 cells led to a significant decrease in cell proliferation, migration, invasion and the IC_50_ value of paclitaxel and adriamycin, while induced cell cycle arrest and apoptosis. And these changes were correlated with down-regulation of Notch signaling, CD44 and cellular prion protein (PrPc). In clinical breast cancer cases, a high ADAM10 expression in pre-NACT samples was strongly associated with poorer response to NACT and shorter overall survival.

**Conclusions:**

These data suggest the previously unrecognized roles of ADAM10 in contributing to the progression and chemo-resistance of TNBC.

## Background

A Disintegrin and Metalloproteinase 10 (ADAM10) is a member of ADAM sheddases which belong to the metzincin superfamily of matrix metalloproteinases (MMPs). The protein structure of ADAM10 contains an N-terminal signal sequence, a catalytic MMP domain, a disintegrin domain related to cell adhesion, an EGF-like domain, a transmembrane domain, and a cytoplasmic tail involved in activity regulation [1, 2]. ADAM10 proteolytically cleaves the ectodomain of a great many transmembrane proteins, including growth factors, cytokines and adhesion molecules, allowing them to transport via a soluble form to neighboring cells [3]. ADAM10 is involved in various physiological processes, such as in activation of the Notch signaling pathway during development [4, 5] and in inflammation/immune response [6, 7]. Dysregulation of ADAM10 activity is associated with pathological processes in some human diseases especially in brain disorders [8]. For example, the upregulation of ADAM10 causes the α-secretase cleavage of amyloid precursor protein (APP) and has been considered as a useful therapeutic approach in Alzheimer’s Disease [9]. The cellular prion protein (PrPc) as a glycosylphosphatidylinositol (GPI)-anchored protein is shed at the plasma membrane by ADAM10 [10], while a pathological and misfolded form of the prion protein (PrPsc) is formed in prion disease. Therefore, activation of ADAM10 aiming at increasing normal PrP breakdown and depleting PrPsc could be seen as putative therapeutic strategy for prion disease [11].

Meanwhile, ADAM10 has been implicated in several types of tumors in humans. ADAM10-mediated Notch1 cleavage and shedding in T-cells controls T-cell development [12], and also contributes to oncogenic Notch signaling by shedding Notch1 mutants in T-cell acute lymphoblastic leukemia (T-ALL) [13]. Constitutive activation of ADAM10 contributes to the growth of mantle cell lymphoma (MCL) cells [14]. Therefore, inhibition of ADAM10 may be a useful strategy to counteract increased Notch1 signaling in T-ALL or to enhance the response of MCL to other therapeutic agents. ADAM10 overexpression in colon cancer cells induces liver metastasis by enhancing L1-CAM cleavage [15], and is correlated with a higher clinical stage in colorectal cancer patient samples [16]. Increase of ADAM10 expression is also found in oral squamous cell carcinoma [17] and pancreatic carcinoma [18]. N-cadherin cleavage is regulated by a protein kinase C-alpha-ADAM10 cascade in glioblastoma (GBM) cells and may mediate GBM cell migration [19].

Recently, ADAM10 has been investigated in breast cancer, particularly the human epidermal growth factor receptor 2 (HER2) enriched subtype. In vitro studies, ADAM10 is identified as one of the major proteases to cleave and shed the HER2 receptor ectodomain [20]. Additionally, ADAM10 contributes to HER receptor activation by shedding of HER ligands such as betacellulin, and it also mitigates Trastuzumab treatment resistance [21]. Zheng et al. has also demonstrated that HER2 extracellular domain (ECD) shedding is associated with α-secretase activity of ADAM10 in breast cancer tissues and cell lines [22]. The depletion of MEL-18 promoted trastuzumab resistance by increasing ADAM10/17 mediated ErbB ligand production and receptor heterodimerization in HER2 positive breast cancer patients [23]. These findings indicate that a combination treatment of ADAM10 inhibitor plus trastuzumab may overcome trastuzumab resistance. However, ADAM10 is rarely studied in triple-negative breast cancer (TNBC) cases.

TNBC is defined by the expression of 1% or less of estrogen receptor (ER) and progesterone receptor (PR) in the tumor, along with the absence of HER2 amplification. TNBC accounts for 15–20% of all breast cancers and occurs generally in younger women, characterized by higher rates of relapse, greater metastatic potential, and shorter overall survival compared with other breast cancer subtypes. Chemotherapy remains the standard treatment for TNBC patients in both the early and advanced-stages of the disease. Identification of biomarkers in TNBC becomes urgent for helping guide treatment decisions [24]. High levels of ADAM10 mRNA were correlated with a poor prognosis in the basal subtype of breast cancer, and knockdown of ADAM10 expression decreased migration in TNBC cell lines [25]. But the mechanism of ADAM10 functions in TNBC cells and its relationship with chemotherapy is still unknown.

In the present study, we have found that the active form of ADAM10 is highly expressed in TNBC cell lines compared with ER + cell line. Knockdown of ADAM10 expression significantly inhibits migration, invasion, and cell growth. It promotes drug susceptibility, and induces cell-cycle arrest and apoptosis of MDA-MB-231 cells via regulating the Notch1 signaling pathway, CD44 and PrPc. Moreover, a study of clinical breast cancer patients receiving neoadjuvant chemotherapy treatment (NACT) demonstrates that higher ADAM10 protein level before NACT was associated with poor response to NACT and shorter overall survival. In conclusion, our results suggest that ADAM10 is involved in the progression and drug-resistance of TNBC, and may be used as a biomarker to develop strategies for treatment.

## Materials and methods

### Cell culture

The human mammary epithelial cell line MCF10A and human breast cancer cell lines MCF7, T-47D, SK-BR-3, MDA-MB-231, MDA-MB-468 and BT-549 were bought from ATCC (Rockville, MD, USA). MCF10A was cultured in MEGM with100 ng/ml cholera toxin (ATCC, USA). MCF7, SK-BR-3 and MDA-MB-468 were cultured in DMEM with 10% fetal bovine serum (FBS, Biowest). T-47D and BT-549 were cultured in RPMI 1640 with 10% FBS. MDA-MB-231 was cultured in L-15 with 10% FBS. All cell lines were cultured at 37 °C in a humidified atmosphere containing 5% CO_2_.

### Quantitative real-time PCR assay (qRT-PCR)

Trizol reagent (Sangon, Shanghai, China) was used to extract total RNA from different cells following the manufacturer’s instructions. Semiquantitative reverse transcription-PCR (RT-PCR) was performed using the one-step RT-PCR kit (Takara, Japan). QRT-PCR assay was carried out in 20 μL solution with 100 ng cDNA and 0.2 μM of each forward and reverse primer and 10 μL of 2 × SYBR green Mix (Takara, Japan). Samples were run using the StepOnePlus real-time PCR system (ABI). The following primers were used for the specific amplification of GAPDH, ADAM10, NOTCH1, CD44 and PRNP: GAPDH forward primer: 5′-CATCAAGAAGGTGGTGAAGC-3′, and reverse primer: 5′-GGAAATTGTGAGGGAGATGC-3′; ADAM10 forward primer: 5′-GCAGACTCGTGGGAAGTTGT-3′, and reverse primer: 5′-ACAGGACACAGGAAGAACCG-3′; NOTCH1 forward primer: 5′-GGACGTCAGACTTGGCTCAG-3′, and reverse primer: 5′-ACATCTTGGGACGCATCTGG-3′; CD44 forward primer: 5′-CAGCAACCCTACTGATGATGACG-3′, and reverse primer: 5′-GCCAAGAGGGATGCCAAGATGA-3′; PRNP forward primer: 5′-AGTGGAACAAGCCGAGTAAGC-3′, and reverse primer: 5′-GTCACTGCCGAAATGTATGATG-3’.

### Western blot analysis and co-immunoprecipitation

Total protein was extracted from cells using RIPA lysis buffer. Cytoplasmic and nuclear proteins were extracted from cells using NE-PER Nuclear and Cytoplasmic Extracion Reagents (Thermo, USA). For Western blots, 30 μg protein extracts were electrophoresed, transferred to PVDF (Millipore, USA) membranes, blocked in 5% non-fat milk for 2 h, and incubated overnight with antibodies against ADAM10 (ab1997, Abcam, USA), PrPc (P0110, Sigma, USA), CD44 (3570, Cell Signaling Technology, USA), Notch1(3608, Cell Signaling Technology, USA), Cleaved-Notch1 (4147, Cell Signaling Technology, USA), Cyclin D3 (2936, Cell Signaling Technology, USA), *p*21 Waf1/Cip1 (2947, Cell Signaling Technology, USA), HES1 (11988, Cell Signaling Technology, USA) and c-Myc (13987, Cell Signaling Technology, USA), GAPDH (sc-47724, Santa Cruz, USA), TBP (sc-74596, Santa Cruz, USA), respectively. And then membranes were incubated for 1 h at room temperature with the appropriate HRP-conjugated secondary antibodies (Proteintech, USA). Detection of protein expression levels by enzyme-linked chemiluminescence (ECL; Pierce, Rockford, USA) was performed according to the manufacturer's protocol.

For co-immunoprecipitation, cells were collected, washed with PBS, and lysed in TNT buffer supplemented with 1 tablet/50 mL of Complete Protease Inhibitor Cocktail (Roche Molecular Biochemical). Lysates were cleared by centrifugation (10,000 × *g* for 15 min at 4 °C) and incubated on ice for 2 h with 10 μg anti-ADAM10, anti-CD44 or anti-PrPc. The antigen sample/antibody mixture was added to a 1.5 mL microcentrifuge tube containing pre-washed Protein G Magnetic Beads (Pierce, Germany) and incubated at room temperature for 1 h with mixing. The beads were retrieved by centrifugation and washed (by vortex and short spin) three times with Wash Buffer. Proteins bound to the beads were eluted by boiling in 2 × electrophoresis sample buffer. Then Western blots were performed as described above.

### Inhibition of ADAM10 expression by RNA interference

2 × 10^5^ cells per well in 2 ml antibiotic-free normal growth medium supplemented with FBS, were seeded in 6-well plates in triplicates. After an overnight incubation, the cells were transfected with different dilutions of siRNA using transfection Reagent (sc-29528, Santa Cruz, USA) as suggested by the manufacturer’s instructions. The small interference RNA (sc-41410, Santa Cruz, USA) was used to target ADAM10 mRNA sequence, while control siRNA (sc-37007, Santa Cruz, USA) was used as negative control. After 24, 48 or 72 h, total RNA was extracted and RT-PCR was performed. Real-time PCR was carried out to detect the mRNA of ADAM10. At 48 or 72 h after transfection, total protein was extracted and protein expression was determined by Western blot.

### Cell migration and invasion assays

The migration and invasion assays of cells were performed as previously described [26], using transwell chambers with 8-μm pore size membranes (Corning Costar, USA) without or with Matrigel (BD Biosciences, San Jose, USA).

### Cell proliferation assay and drug sensitivity assay

Cell proliferation was assessed using CCK8 (Dojindo, Tokyo, Japan). The cells were seeded on 96-well microplates at a density of 5 × 10^3^ cells per well. At 0–4 days after transfection with ADAM10 siRNA, the cells were incubated with 10 μl of CCK8 for 3 h. Then the OD of each sample was measured at a 450 nm test wavelength with an ELISA multi-well spectrophotometer (Molecular Devices Corp., Sunnyvale, CA, USA).

For drug sensitivity assay, cells transfected with ADAM10 siRNA or negative control siRNA were seeded in 96-well plates at a density of 3 × 10^4^ cells per well and incubated with serially diluted paclitaxel (0, 2, 4, 6 and 8 μg/ml), or adriamycin (0, 0.2, 0.4, 0.6 and 0.8 μg/ml) for 24 h followed by 2 h incubation with CCK-8 solution. The OD of each well was measured at a 450 nm test wavelength. The cell survival rate was calculated based on the OD of the negative control cells. The 50% inhibitory concentration (IC50) values were determined as the drug concentration causing 50% cell growth inhibition.

### Flow cytometry analysis

For cell cycle analysis, cells were harvested, washed with PBS, and fixed. Prior to the analysis, the cells were incubated with fresh propidium iodide containing RNase for 30 min at 37 °C. DNA content was determined by fluorescence-activated cytometry (FACS) analysis of the propidium iodide-stained cells using a FACSCalibur flow cytometer (BD Biosciences, San Jose, CA, USA).

Apoptosis analysis was carried out by dual dye staining using Annexin V and 7-AAD. Cells were harvested, washed twice with PBS, and stained with PE Annexin V apoptosis detection kit according to the manufacturer’s instructions. The stained cells were subjected to a FACSCalibur flow cytometer and the results were analyzed using the Flow Jo 7.6.1 software (Tree Star Inc., USA).

### Patient characteristics and immunohistochemistry

Paraffin-embedded tissue samples from 94 primary breast cancer patients, which were diagnosed as “invasive carcinoma of no special type” at the Department of Pathology, Huashan Hospital of Fudan University between 2011 and 2013, were collected. Prior to radical mastectomy, the patients received neoadjuvant chemotherapy treatment (NACT) with cyclophosphamide, epirubicin/epidoxorubicin and taxol combination therapy at the Department of Breast Surgery in Huashan Hospital. These patients were graded into G1–G5 based on the Miller-Payne grading system [27] according to their response to NACT, which were evaluated by two pathologists (Tang F and Bao Y). Then the 94 cases were divided into two cohorts, including Cohort 1 (G1 and G2) with poor response to NACT and Cohort 2 (G3, G4 and G5) with good response to NACT. From each patient, a core-needle biopsy of the tumor was taken before NACT and a post-NACT radical mastectomy was excised. The specimens were paraffin-embedded for subsequent immunohistochemical staining. Slides were dehydrated in xylene and graded alcohols. Antigen retrieval was performed with 0.01 M citrate buffer at pH 6.0 at 95 °C for 10 min. Then slides were incubated with diluted primary antibody anti-ADAM10 (sc-48400, Santa Cruz, USA) in 1:100 dilution for 12 h followed by incubations with biotinylated secondary antibody for 1 h, and diaminobenzidine (DAKO, Denmark) for 10 min. Slides were again counterstained with Mayer’s hematoxylin.

The saturation and intensity of the immunostained cells were evaluated over three visual fields, at a power of × 200 under a light microscope (Nikon, Japan). ADAM10 immunoreactivity was detected mainly in the cytoplasm and cytomembrane. According to H scoring system [28], the total staining of ADAM10 was based on the intensity score (0, 1, 2, 3) multiplying the percentage of positive cells, giving a possible range of 0–300 (%). Then ADAM10 low expression group ranged from 0 to 200 (including 200), and the high expression group ranged from 200 to 300.

### Statistical analyses

Statistical analyses were performed using IBM SPSS, version 21.0. All experiments were repeated at least three times and the results are presented with mean ± standarderrors (SEM). The differences were analyzed by using the Student’s t-test. To correlate ADAM10 expression with clinicopatholgical factors, we used Chi-Square or Mann–Whitney U tests, respectively, for categorical and non-categorical variables. Correlation analysis was performed using Spearman’s rank correlation coefficients. In multivariate analyses, all the data sets were pooled and the odds ratios and *P* values were estimated with logistic regression model stratified by study. Overall survival was calculated using the Kaplan–Meier analysis and differences between groups were assessed using log-rank tests. Univariate and multivariate Cox regression analysis was performed to evaluate differences of clinicopatholgical factors in the risk of death. For all tests, a *p* < 0.05 (two-tailed) was defined as statistically significant.

## Results

### The active form of ADAM10 is highly expressed in triple-negative breast cancer cell lines

**Fig. 1:**
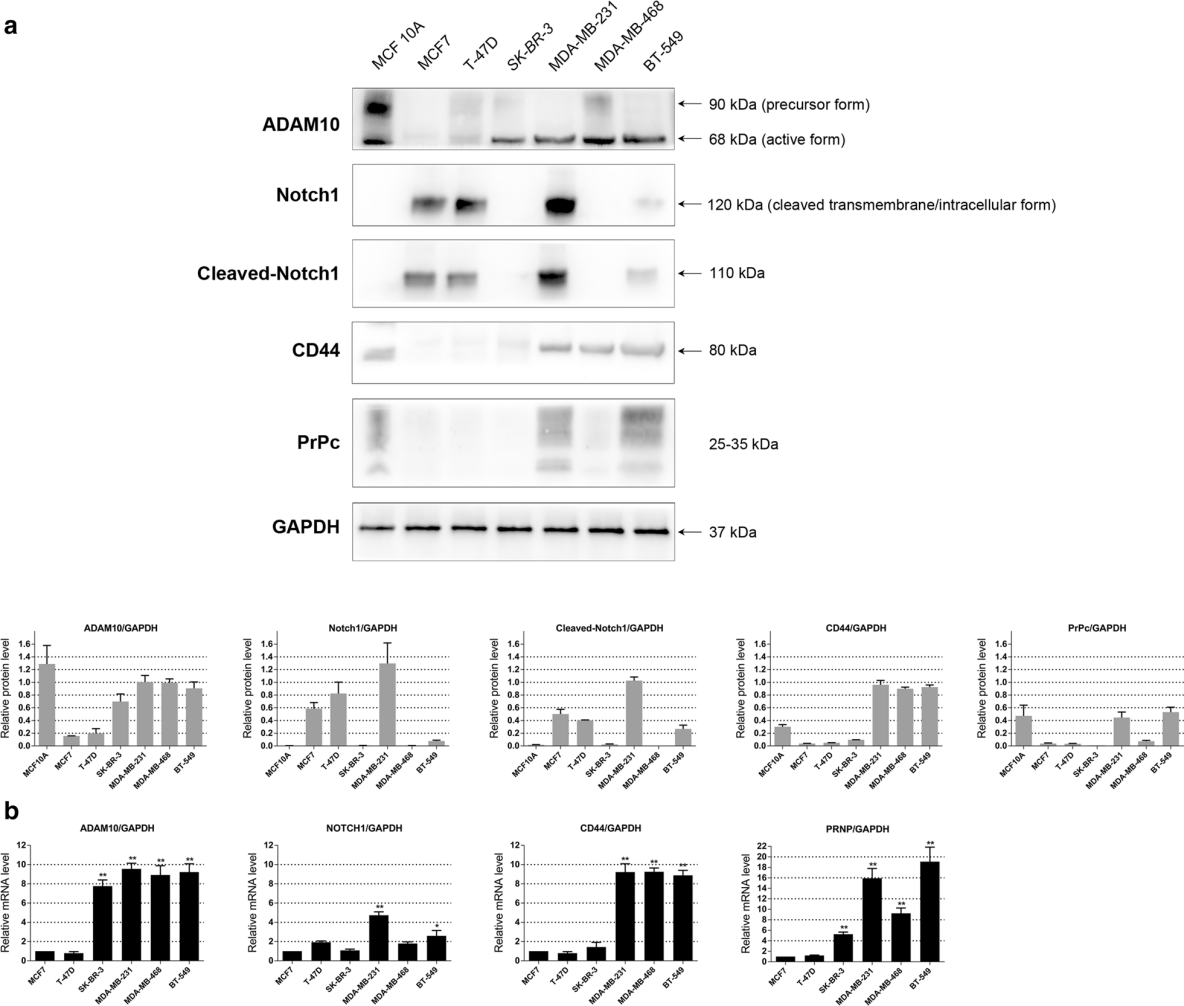
ADAM10, Notch1, Cleaved-Notch1, CD44 and PrPc expression in MCF10A and different breast cancer cell lines. **a** Western blot assay showed protein expression level of ADAM10, Notch1, Cleaved-Notch1, CD44 and PrPc in human mammary epithelial cell line MCF10A and different breast cancer cell lines MCF7, T-47D, SK-BR-3, MDA-MB-231, MDA-MB-468 and BT-549. GAPDH was used as a loading control. Quantitative analysis of band intensities was conducted in western blot analyses. Data are shown as mean ± SEM of three independent experiments. **b** QRT-PCR assay showed mRNA expression level of ADAM10, Notch1, CD44 and PrPc in different breast cancer cell lines. GAPDH was used as a loading control. Bars represent the mean of triplicate samples; error bars represent SD. Data are representative of three independent experiments. The significant difference between MCF7 and all the other cell line is indicated by *p < 0.05, **p < 0.01

First, we analyzed the protein expression level of ADAM10 in human mammary epithelial cell line MCF10A and different breast cancer cell lines MCF7, T-47D, SK-BR-3, MDA-MB-231, MDA-MB-468 and BT-549 using the western blot assay. The active form at 68 kDa of ADAM10 was highly expressed in triple-negative cell lines MDA-MB-231, MDA-MB-468 and BT-549 and HER2 amplified cell line SK-BR-3. On the other hand, both the precursor form at 90 kDa and active form at 68 kDa of ADAM10 were detectable in MCF10A, while barely detectable in ER + cell lines MCF7 and T-47D (Fig. 1a). The mRNA expression level of ADAM10 was also higher in triple-negative cell lines and HER2 amplified cell line compared to ER + cell lines using qRT-PCR assay (Fig. 1b). These results suggest that ADAM10 in its active form is highly expressed in triple-negative cell lines.

### Down-regulation of ADAM10 affects the functions of triple-negative breast cancer cells

**Fig. 2:**
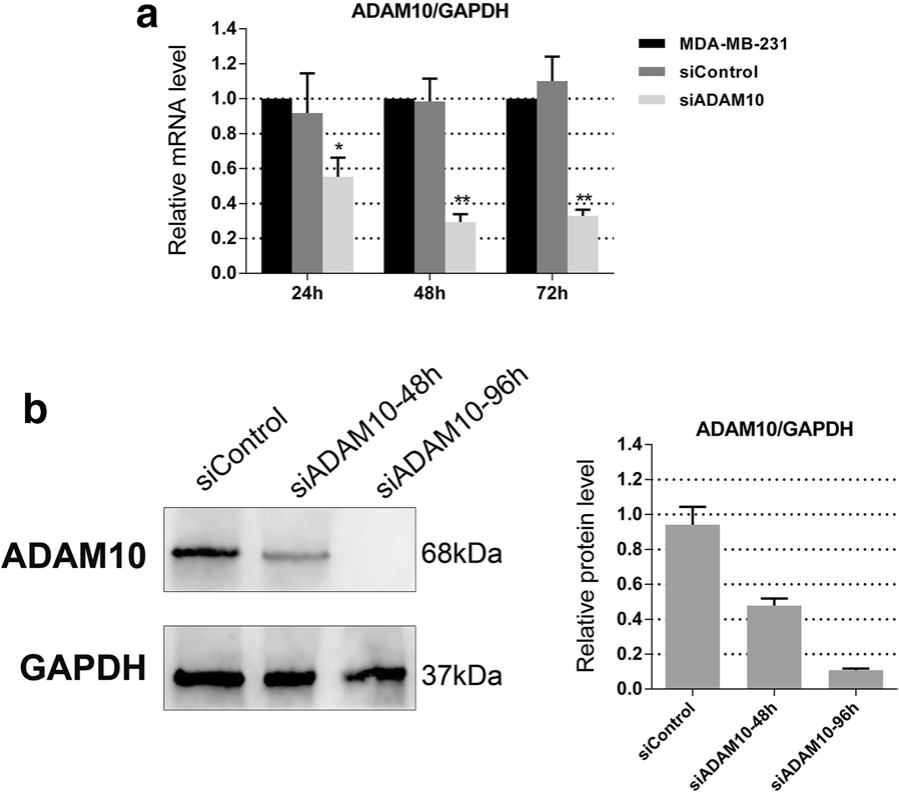
ADAM10 is down-regulated in MDA-MB-231 cells with the use of ADAM10 siRNA. **a** Knockdown efficiency was determined by qRT-PCR after 24, 48 and 72 h transfecting ADAM10 siRNA in MDA-MB-231 cells. GAPDH was used as a loading control. Bars represent the mean of triplicate samples; error bars represent SD. Data are representative of three independent experiments. The significant difference between MDA-MB-231 and MDA-MB-231 with siControl or siADAM10 is indicated by *p < 0.05, **p < 0.01. **b** Knockdown efficiency was determined by western blot after 48 and 96 h transfecting ADAM10 siRNA in MDA-MB-231 cells. GAPDH was used as a loading control. Quantitative analysis of band intensities was conducted in western blot analyses. Data are shown as mean ± SEM of three independent experiments

**Fig. 3:**
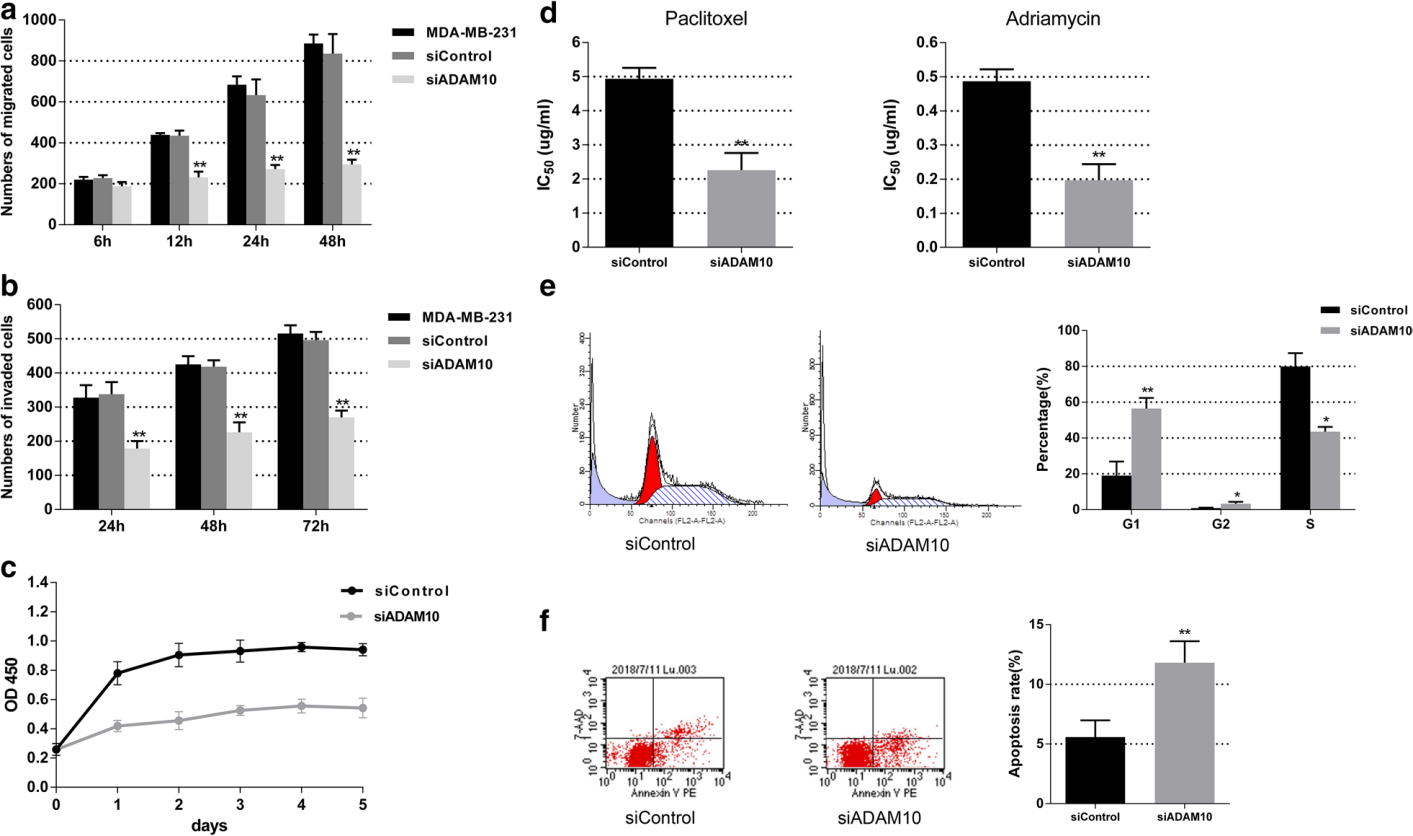
Effects of ADAM10 down-regulation are detected in ADAM10 knockdown MDA-MB-231 cells. Knockdown of ADAM10 expression in MDA-MB-231 cells attenuated the migration (**a**) and invasion (**b**) ability. **c** CCK8 assay was used for detection of proliferation in MDA-MB-231 cells with siADAM10 or siControl. **d** The 50% inhibitory concentration (IC_50_) value of paclitaxel and adriamycin were tested in MDA-MB-231 cells with siADAM10 or siControl. **e** Cell-cycle analysis by flow cytometry using propidium iodide showed significant G1/2 cell-cycle arrest in ADAM10 knockdown MDA-MB-231 cells compared to siControl. **f** The apoptotic cells were detected by PE Annexin V and 7-AAD. Cells that are considered viable are PE Annexin V negative and 7-AAD negative; cells in early apoptosis are PE Annexin V positive and 7-AAD negative; and cells in late apoptosis or already dead are both PE Annexin V and 7-AAD positive. Bars represent the mean of triplicate samples; error bars represent SD. Data are representative of three independent experiments. The significant difference between siControl and siADAM10 is indicated by *p < 0.05, **p < 0.01

To examine the function of ADAM10 in triple-negative breast cancer, we chose the triple-negative cell line MDA-MB-231 for further study. After siRNA knockdown of ADAM10 in MDA-MB-231 cells, both ADAM10 mRNA and the active form of ADAM10 protein were significantly reduced in comparison to the siControl (Fig. 2a and b). Then we performed a transwell chamber assay without or with matrigel in ADAM10 knockdown MDA-MB-231 cells, and the migration and invasion ability was dramatically decreased (Fig. 3a and b). The knockdown of ADAM10 expression in MDA-MB-231 cells also suppressed cell proliferation compared with the siControl using CCK8 assay (Fig. 3c). Similar to the results in MDA-MB-231, knockdown of ADAM10 expression in a different TNBC cell line BT-549, noticeably attenuated cell migration, invasion and proliferation abilities (Additional file 1: Figure S1). On the other hand, the IC_50_ value of paclitaxel and adriamycin in MDA-MB-231 cells with siADAM10 was significantly lower than the siControl (Fig. 3d).

Knockdown of ADAM10 using siRNA in mantle cell lymphoma cells induces cell-cycle arrest but not apoptosis [14]. In order to verify whether ADAM10 is involved in the cell cycle and apoptosis of TNBC cells, we performed cell-cycle analysis on MDA-MB-231 cells. Compared with cells transfected with the negative control siRNA, transfection of ADAM10 siRNA in MDA-MB-231 induced a significant cell-cycle arrest at the G1/G2 phase and reduced the proportion of cells in the S phase (Fig. 3e). The apoptotic cells were detected by PE Annexin V and 7-AAD. The proportion of apoptotic cells in the siADAM10 group was higher than that of the siControl group (*p* < 0.01) (Fig. 3f). As a result, knockdown of ADAM10 by siRNA in TNBC cells significantly reduces cell migration, invasion and cell growth. It also induces cell-cycle arrest, causes cell apoptosis, and promotes drug susceptibility.

### ADAM10 affects the functions of triple-negative breast cancer cells via regulating Notch1 signaling pathway

**Fig. 4:**
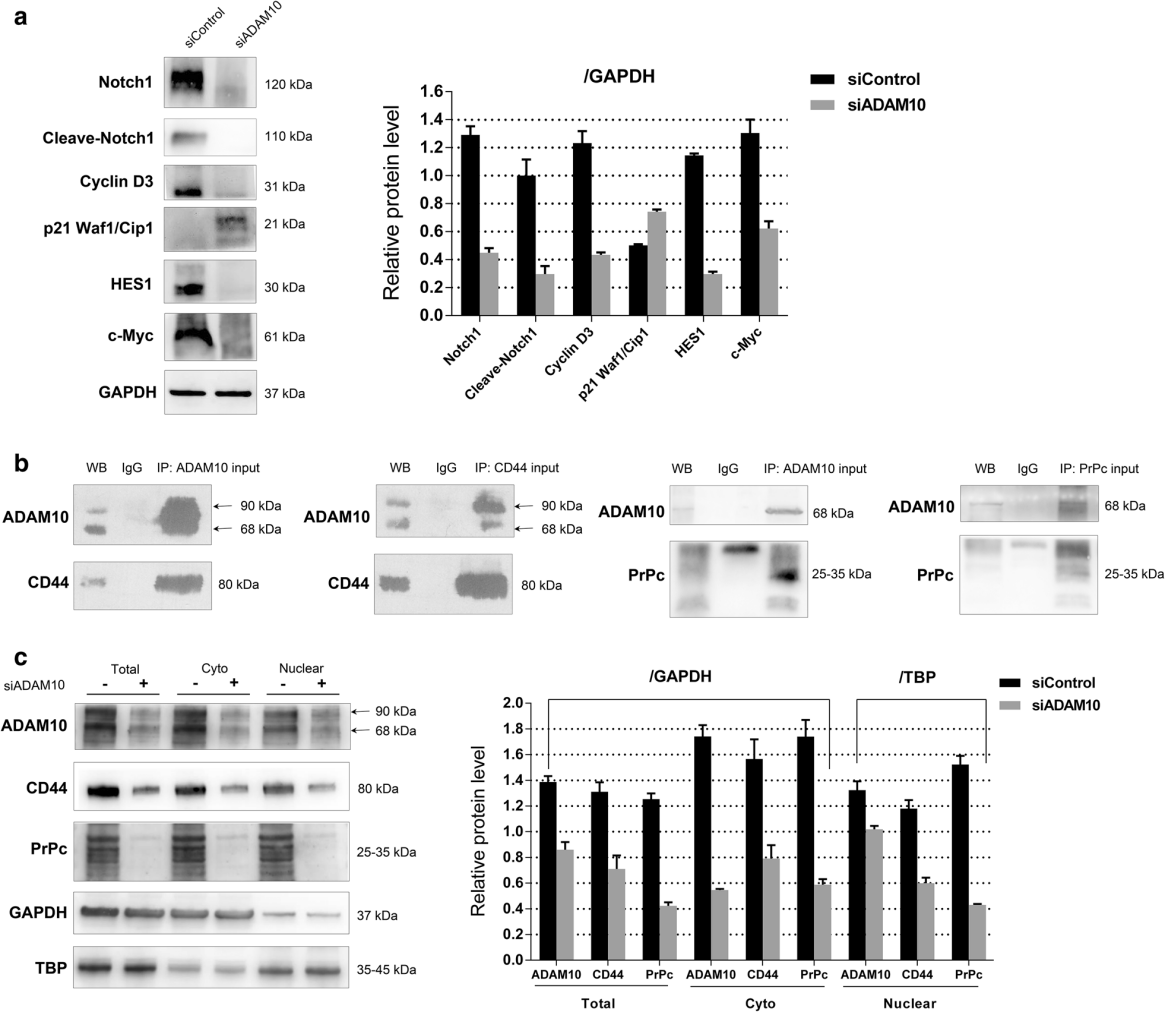
ADAM10 affects the functions of MDA-MB-231 cells via regulating Notch signaling pathway, CD44 and PrPc. **a** Western blot assay showed Notch1, Cleave-Notch1 and its activated targets Cyclin D3, *p*21Waf1/Cip1, HES1 and c-Myc expression in MDA-MB-231 cells with siADAM10 or siControl for 96 h. GAPDH was used as a loading control. **b** WB lane stands for lysates from MDA-MB-231 cells immunoblotted with corresponding antibodies without IgG, ADAM10, CD44 or PrPc antibody incubation. IgG lane stands for lysates immunoblotted with corresponding antibodies after IgG incubation. IP lane stands for lysates immunoprecipitated with ADAM10, CD44 or PrPc antibody and immunoblotted with corresponding antibodies. **c** Cytoplasmic and nuclear extracts from MDA-MB-231 cells with siADAM10 or siControl were tested by Western blot assay, showing protein expression level of ADAM10, CD44 and PrPc. GAPDH was used as a cytoplasmic control and TBP as a nuclear control. Quantitative analysis of band intensities was conducted in western blot analyses. Data are shown as mean ± SEM of three independent experiments

Notch signaling plays important roles in sustaining proliferative signaling, protecting from apoptosis, reducing the chemoresistance and controlling the cancer stemness [29]. Among the four Notch receptors (Notch1-4), the Notch1 receptor has been reported as one potential oncogenic activator and over-expressed in TNBC [30]. ADAM-mediated proteolysis is required for Notch receptor activation [31]. To demonstrate whether ADAM10 affects TNBC functions by regulating the Notch1 signaling, we first detected the expression level of Notch1 in different breast cancer cell lines using the western blot and qRT-PCR assays. The cleaved transmembrane/intracellular form of Notch1 was highly expressed in MDA-MB-231 cells (Fig. 1a and b). After siRNA knockdown of ADAM10 in MDA-MB-231 cells, Notch1, cleaved Notch1 and its activated targets Cyclin D3, HES1 and c-Myc were down-regulated, whereas *p*21 Waf1/Cip1 was up-regulated (Fig. 4a). These results indicate that knockdown of ADAM10 by siRNA in TNBC cells inhibits cell proliferation by regulating Notch1, HES1 and c-Myc, and induces cell-cycle arrest by regulating Cyclin D3 and *p*21 Waf1/Cip1.

### ADAM10 interacts with CD44 and PrPc and promotes their nuclear transportation

ADAM10 is a major protease acting on CD44 intracellular domain phosphorylation [32], and contributes to the cleavage of PrPc in human HEK293 cells [10]. In TNBC, CD44 + /CD24- phenotypes are associated with cancer stem cells [33]. In our previous study, PrPc was demonstrated to interact with P-gp [34] and CD44 [26] to promote multidrug resistance in adriamycin-resistant breast cancer cell line MCF7/ADR. In the present study, knockdown of ADAM10 in MDA-MB-231 cells could significantly reduce the IC_50_ value of paclitaxel and adriamycin (Fig. 3d). Then, we speculate that ADAM10 may promote drug resistance by regulating CD44 and PrPc in TNBC cells. We first tested CD44 and PrPc expression level in different breast cancer cell lines using the western blot and qRT-PCR assays. In comparison to MCF7, CD44 and PrPc were highly expressed in TNBC cell lines (Fig. 1a and b). Then we performed a co-immunoprecipitation assay using cell lysates from MDA-MB-231 cells. The immunoprecipitation with anti-ADAM10 coprecipitated CD44 and PrPc, and the immunoprecipitation with anti-CD44 or anti-PrPc also coprecipitated ADAM10 in the cell lysates (Fig. 4b). Furthermore, both cytoplasmic and nuclear CD44 and PrPc protein levels in ADAM10 knockdown MDA-MB-231 cells were decreased (Fig. 4c). Together, these data show that ADAM10 interacts with CD44 and PrPc, and initiates CD44 and PrPc intramembrane proteolysis followed by nuclear transport and signaling of the cytoplasmic domain.

### High ADAM10 expression level is associated with poor response to neoadjuvant chemotherapy treatment in breast cancer patients

**Fig. 5:**
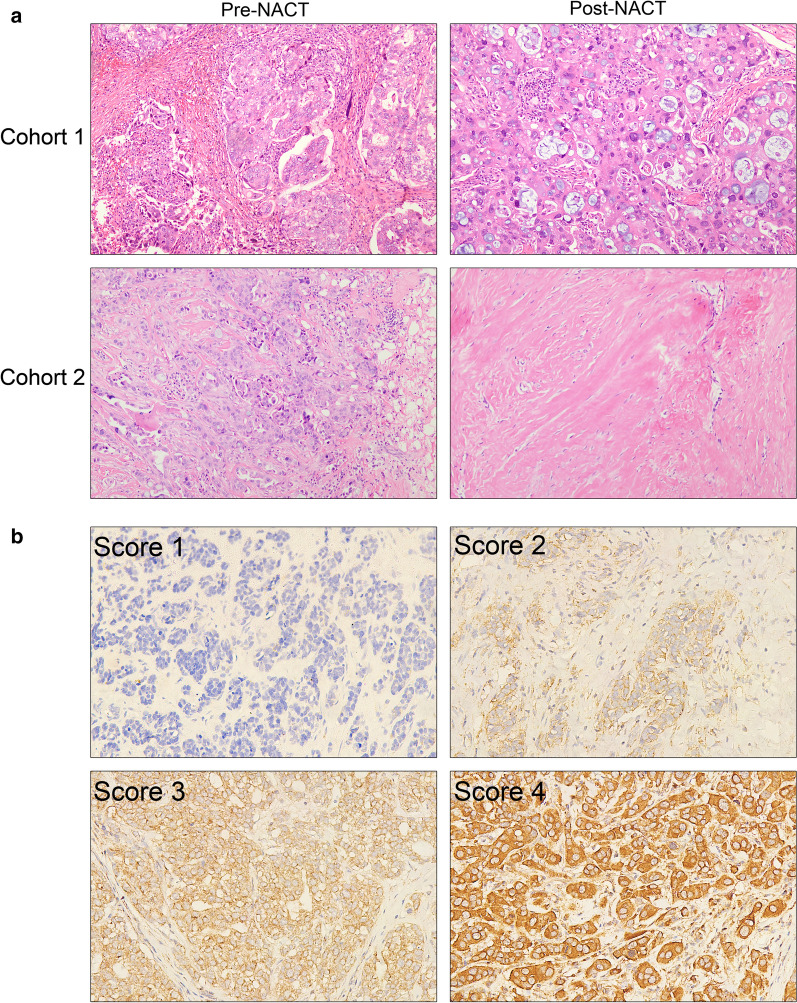
Representative H&E-stained samples and immunohistochemical staining of ADAM10. **a** Two representative H&E-stained samples were diagnosed as invasive breast carcinoma of no special type and classified into Cohort 1 and Cohort 2, respectively, according to their different responses to neoadjuvant chemotherapy (NACT). Cohort 1: H&E-stained sample before and after NACT showed poor response to NACT. Cohort 2: H&E-stained sample before and after NACT showed good response to NACT. **b** Representative immunohistochemical staining intensity of ADAM10 scaled 0, 1, 2, 3 (Score 0: negative staining; Score 1: weak staining; Score 2: moderate staining; Score 3: strong staining). The positive staining of ADAM10 was detected in cytoplasm and cytomembrane. All representative images were taken on power of × 200

As described in Fig. 3d, knockdown ADAM10 could reduce the IC_50_ value of paclitaxel and adriamycin in MDA-MB-231 cells. We speculate that ADAM10 is correlated with chemotherapeutic effects in breast cancer. So we selected tissue samples from two cohorts of breast cancer patients who had distinctly different responses to neoadjuvant chemotherapy treatment (NACT) (Fig. 5a). The patients’ characteristics are described in the “Materials and Methods” section. Immunohistochemical staining of ADAM10 was performed on the tissues from a core-needle biopsy of the tumor before NACT and a post-NACT radical mastectomy in these two cohorts of breast cancer patients. The ADAM10 immunoreactivity was detected mainly in the cytoplasm and cytomembrane (Fig. 5b).

In Cohort 1 patients with poor response to NACT, the ADAM10 expression of pre-NACT samples was higher than that of post-NACT samples (*p* = 0.039). Conversely, patients in Cohort 2 who had a good response to NACT, the ADAM10 expression of pre-NACT samples was lower than that of post-NACT samples (*p* = 0.030). Moreover, we observed that ADAM10 protein level of pre-NACT samples was higher in Cohort 1 than that of Cohort 2 (*p* = 0.023), and the ADAM10 protein level of post-NACT samples was lower in Cohort 1 than that of in Cohort 2 (*p* = 0.020) (Table 1).

**Table 1:** ADAM10 expression level before and after neoadjuvant chemotherapy (NACT) in Cohort 1 and Cohort 2 according to different chemotherapy response

	Pre-NACT (mean ± SE)	Post-NACT (mean ± SE)	*p*-value
Cohort 1 (n = 25)	172.4 ± 77.18	118.4 ± 86.98	0.039*
Cohort 2 (n = 69)	129.1 ± 88.04	170.6 ± 94.49	0.030*
*p*-value	0.023*	0.020*	

Cohort 1: Samples showing poor response to NACT

Cohort 2: Samples showing good response to NACT

*p < 0.05, **p < 0.01

Afterwards we examined the clinical value of pre-NACT ADAM10 expression in the 94 breast cancer cases using univariate and multivariate analyses (Table 2). In multivariate logistic regression analysis, high ADAM10 expression was correlated with poor responses to NACT (OR = 0.29, *p* = 0.042), and lower BMI (OR = 0.17, *p* = 0.002). Then we analyzed the correlation between the response to NACT and different clinicopathological factors (Table 3). In multivariate logistic regression analysis, the response to NACT only significantly correlated with pre-NACT ADAM10 expression level (OR = 0.25, *p* = 0.028). It showed that poor response to NACT correlated with high ADAM10 expression. These results suggest that the high expression level of ADAM10 in breast cancer is linked to poor response to clinical chemotherapies.

**Table 2:** Correlation between ADAM10 expression and clinic pathological factors in 94 breast cancer tissues

Factors	ADAM10 expression			Univariate analysis		Multivariate analysis	
	Low (mean ± SE)	High (mean ± SE)	*p*-value	OR (95%CI)	*p*-value	OR (95%CI)	*p*-value
*Age at diagnosis (years)*							
< 55	34 (120.4 ± 54.60)	15 (252.0 ± 34.58)	0.008**	0.35 (0.12–1.00)	0.050	0.96 (0.19–4.77)	0.963
≥ 55	39 (91.2 ± 62.02)	6 (297.5 ± 6.12)					
*Menopause*							
Yes	53 (98.9 ± 62.35)	10 (277.5 ± 27.61)	0.024*	0.34 (0.13–0.93)	0.036*	0.45 (0.05–0.53)	0.292
No	20 (120.5 ± 51.9)	11 (253.6 ± 39.94)					
*BMI (kg/m*^*2*^)							
> 23	51 (106.7 ± 56.50)	6 (245.0 ± 37.55)	0.023*	5.80 (1.99–16.91)	0.001**	0.17 (0.10–2.00)	0.002**
≤ 23	22 (100.5 ± 68.97)	15 (273.0 ± 33.16)					
*Histological grade*							
I	4 (95.0 ± 72.23)	2 (262.5 ± 53.03)	0.172	0.55 (0.18–1.65)	0.286		
II	56 (98.4 ± 59.23)	17 (270.0 ± 34.37)					
III	13 (135.4 ± 55.28)	2 (225.0 ± 21.21)					
*Tumor size*							
T1	4 (78.8 ± 55.43)	2 (262.5 ± 53.03)	0.683	0.89 (0.29–2.76)	0.846		
T2	60 (104.8 ± 62.52)	16 (266.3 ± 37.35)					
T3	9 (116.1 ± 45.26)	3 (260.0 ± 31.22)					
*Distant metastasis (before NACT)*							
Yes	4 (107.5 ± 70.77)	3 (300)	0.287	0.56 (0.06–4.92)	0.599		
No	53 (109.7 ± 63.91)	16 (282.2 ± 21.37)					
*Regional lymphatic metastasis (after NACT)*							
Yes	38 (103.3 ± 62.63)	9 (283.3 ± 21.79)	0.066	1.42 (0.52–3.83)	0.492		
No	19 (122.1 ± 65.77)	10 (286.5 ± 20.55)					
*Response to NACT*							
Good	58 (99.5 ± 59.41)	11 (285 ± 27.66)	0.023*	0.28 (0.10–0.79)	0.016*	0.29 (0.44–16.22)	0.042*
Poor	15 (125.3 ± 60.34)	10 (243.0 ± 31.46)					

*OR* odds ratio, *CI* confidence interval

*p < 0.05, **p < 0.01

**Table 3:** Correlation between different responses to NACT and clinic pathological factors in 94 breast cancer tissues

Factors	Response to NACT			Univariate analysis		Multivariate analysis	
	Poor	Good	*p*-value	OR (95%CI)	*p*-value	OR (95%CI)	*p*-value
*ADAM10 expression level (pre-NACT)*							
Low	15	58	0.013*	0.28 (0.10–0.79)	0.016*	0.25 (0.07–0.86)	0.028*
High	10	11					
*ADAM10 expression level (post-NACT)*							
Low	21	36	0.266	2.19 (0.64–7.46)	0.211		
High	4	15					
*Age at diagnosis (years)*							
< 55	19	30	0.009**	4.12 (1.46–11.58)	0.007**	3.85 (0.99–14.92)	0.051
≥ 55	6	39					
*Menopause*							
Yes	13	50	0.055	2.43 (0.94–6.26)	0.066		
No	12	19					
*BMI (kg/m*^*2*^)							
> 23	14	43	< 0.001**	0.77 (0.30–1.95)	0.580		
≤ 23	26	11					
*Histological grade*							
I	3	3	0.059	0.67 (0.25–1.79)	0.420		
II	15	58					
III	7	8					
*Tumor size*							
T1	2	4	0.772	0.89 (0.31–2.55)	0.827		
T2	19	57					
T3	4	8					
*Distant metastasis (before NACT)*							
Yes	2	5	1.000	0.90 (0.16–4.96)	0.902		
No	23	64					
*Regional lymphatic metastasis (after NACT)*							
Yes	10	32	0.644	0.77 (0.30–1.95)	0.583		
No	15	37					

*OR* odds ratio, *CI* confidence interval

*p < 0.05, **p < 0.01

### ADAM10 expression is an independent prognostic factor in breast cancer

**Fig. 6:**
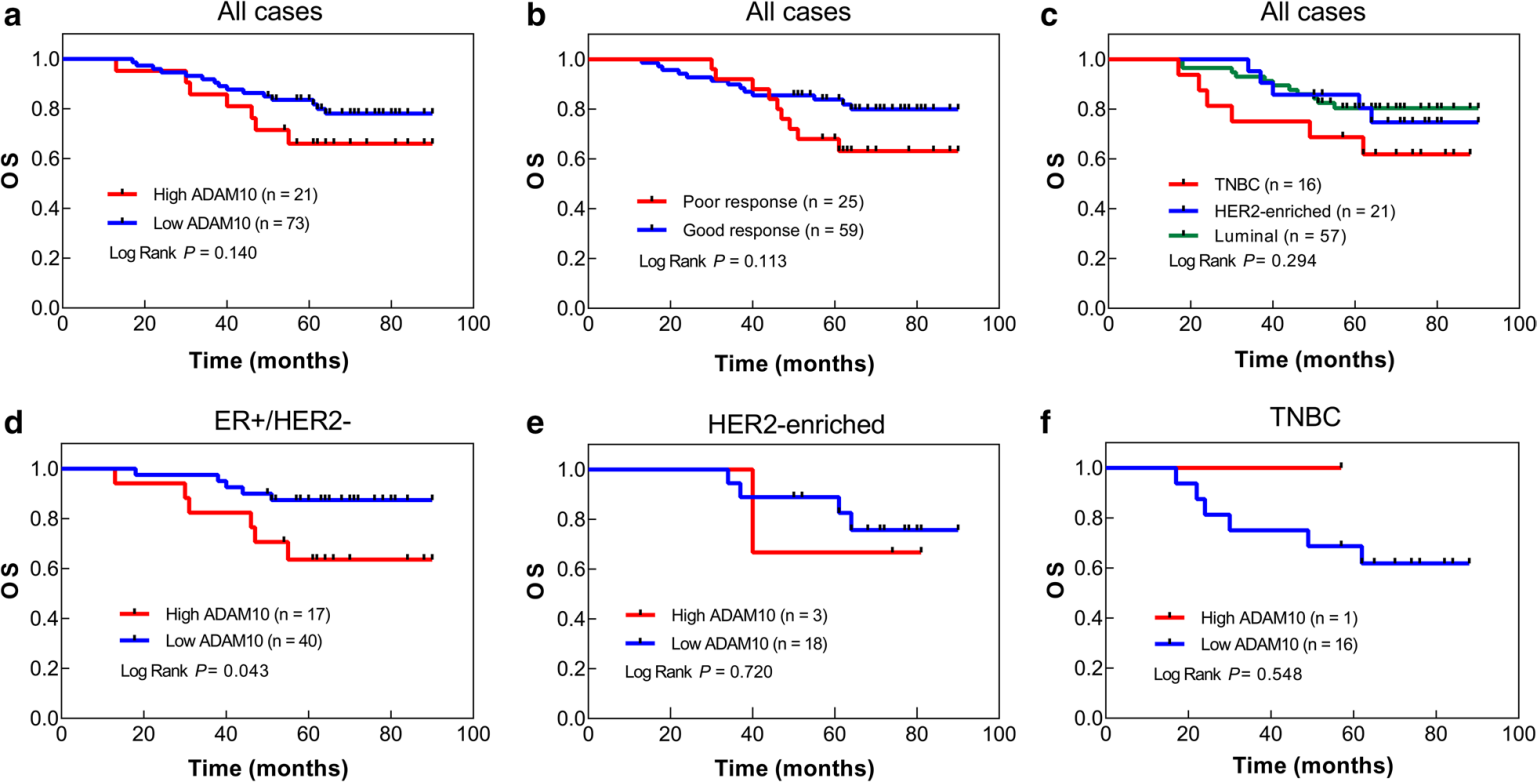
Kaplan–Meier curves of overall survival between different groups in 94 breast cancer patients receiving NACT. Kaplan–Meier plots of overall survival of 94 patients stratified by **a** high/low ADAM10 expression of pre-NACT samples, **b** poor/good response to NACT and **c** different molecular breast cancer subtypes were demonstrated. Kaplan–Meier plots depicting the impact of high ADAM10 expression on overall survival of **d** ER + /HER2− subtype, **e** HER2-enriched subtype and **f** triple-negative subtype. Statistical analyses were performed using log-rank tests

Kaplan–Meier curves were used to analyze overall survival among 94 cases. The first and last follow-ups were carried out in January 2011 and February 2019. Pre- NACT ADAM10 expression level (Fig. 6a), patients’ response to NACT (Fig. 6b) and molecular classification (Fig. 6c) did not have a statistically significant effect on overall survival. Shorter overall survival was observed in patients with high ADAM10 expression in ER + /HER2- subtype (*p* = 0.043) (Fig. 6d), but was not observed in HER2-enriched subtype (Fig. 6e) or TNBC subtype (Fig. 6f).

Univariate and multivariate Cox regression analysis of 94 cases was performed to evaluate differences in the clinicopathological factors for the risk of death (Table 4). In Cox proportional hazards model for multivariate survival analysis, pre-NACT ADAM10 (HR = 3.67, *p* = 0.028), Ki67 index (HR = 3.33, *p* = 0.049), tumor size (HR = 2.84, *p* = 0.045) and lymphatic metastasis (HR = 8.13, *p* = 0.002) had a statistically significant effect on overall survival. The results indicate that the increase of ADAM10 expression is an independent predictor of poor overall survival.

**Table 4:** Cox analyses of overall survival for breast cancer patients receiving NACT

Factors	Univariate analysis		Multivariate analysis	
	*p*-value	HR (95%CI)	*p*-value	HR (95%CI)
ADAM10 (Pre-NACT)	0.147	1.90 (0.80–4.52)	0.028*	3.67 (1.15–11.70)
ER	0.359	0.84 (0.57–1.23)	0.253	0.68 (0.35–1.32)
PR	0.246	0.72 (0.41–1.26)	0.982	0.99 (0.45–2.19)
HER2	0.727	0.86 (0.37–1.99)	0.531	0.73 (0.28–1.94)
Ki67	0.199	1.80 (0.73–4.43)	0.049*	3.33 (1.01–10.99)
Age	0.16	0.54 (0.23–1.28)	0.553	0.74 (0.27–2.01)
BMI	0.35	1.48 (0.65–3.36)	0.716	0.83 (0.31–2.25)
Histological grade	0.830	1.13 (0.38–3.31)	0.112	2.85 (0.78–10.37)
Tumor size	0.002**	4.11 (1.69–10.03)	0.045*	2.84 (1.02–7.88)
Distant metastasis	0.602	0.59 (0.08–4.36)	0.528	0.51 (0.06–4.15)
Lymphatic metastasis	0.011*	3.66 (1.35–9.94)	0.002**	8.13 (2.10–31.43)
Response to NACT	0.082	0.48 (0.21–1.10)	0.993	1.00 (0.38–2.67)

*p < 0.05, **p < 0.01

## Discussion

Breast cancer is the most frequently diagnosed cancer and the second leading cause of cancer deaths in women worldwide. Clinically, this heterogeneous disease is categorized into three basic therapeutic groups: ER positive group, HER2 enriched group and triple-negative group. Triple-negative breast cancer (TNBC) lacks of ER, PR and HER2 expression and generally occurs in younger women. It is characterized by higher rates of relapse, greater metastatic potential, and shorter overall survival with only chemotherapy options. To date, the molecular mechanisms that drive TNBC occurrence have not been fully elucidated. Targeted therapies have not significantly improved survival in patients with TNBC [24, 35].

Previous work has mainly focused on genetic and transcriptional changes in TNBC. Proteolytic cleavage represents a unique and irreversible posttranslational event to regulate the function and half-life of many intracellular and extracellular proteins. ADAM10 belongs to the ADAM family of metalloproteinases which cleave and shed the ectodomain of hundreds of transmembrane proteins, and it plays important roles in physiological and pathophysiologcial processes. It has also been implicated in the pathogenesis of several types of human malignant tumors including breast cancer [36]. However, the studies on the roles of ADAM10 in breast cancer have only focused on the HER2 positive type [20–23] rather than on the triple-negative type [25, 37]. Here, we have demonstrated that the higher level of ADAM10 in its active form is expressed not only in HER2 amplified cell line SK-BR-3 but also in TNBC cell lines MDA-MB-231, MDA-MB-468 and BT-549, compared with ER positive cell line MCF7. Our findings also suggest that ADAM10 is biologically significant in TNBC. ADAM10 promotes migration, invasion, cell growth, cell-cycle progression, and blocks cell apoptosis of TNBC cells, with the activation of the Notch signaling pathway.

The Notch signaling system that contains four Notch receptors (Notch1- Notch4) and five canonical ligands (Dll1, Dll3, Dll4, Jagged1 and Jagged2), plays important roles including carcinogenesis, cancer stem cell renewal, angiogenesis, and chemotherapy resistance in the progression of breast cancer [38]. Notch1 is highly expressed in poorly differentiated breast tumors and associated with poor overall survival [39]. Specifically inhibiting human Notch1 in triple negative breast cancer enhances the antitumor efficacy of chemotherapy and decelerates tumor growth by reducing cancer stem cells [30]. The Notch receptor is activated by ligand binding, followed by ADAM10/ADAM17 mediated proteolytic cleavage at the Notch extracellular domain (NECD) site 2 (S2) [4, 31] and a second γ-secretase proteolytic cleavage at the Notch transmembrane domain (NTM) site 3 (S3). These result in release of the Notch intracellular domain (NICD), which translocates to the nucleus and activates transcription of downstream target genes [40]. Notably, regulation of the Notch signaling by ADAM10 in TNBC has not been reported before. Our study has demonstrated that knockdown of ADAM10 using siRNA decreases the expression of Notch1. The expression level of Notch1 targets Cyclin D3, HES1 and c-Myc is reduced, while *p*21 Waf1/Cip1 is increased. Cyclin D3 regulates the cell cycle by controlling physiological progression from G1 to S phase [41], whereas the tumor suppressor *p*21 Waf1/Cip1 acts as an inhibitor of cell cycle progression [42]. HES1 and c-Myc have been implicated in cancer [43]. Hence, we speculate that ADAM10 promotes cell cycle progression, growth and metastasis of TNBC cells by regulation of Notch1and its targets Cyclin D3, *p*21 Waf1/Cip1, Hes1 and c-Myc.

Due to lack of targeted therapies in TNBC, chemotherapy remains the standard of care. Chemotherapeutic drugs for breast cancer include epirubicin, doxorubicin, paclitaxel, and docetaxel, each aiming at blocking the proliferation of breast cancer cells. However, consequent chemo-resistance results in the failure of chemotherapy and tumor relapse. In our study, knockdown of ADAM10 could strengthen the sensitivity of chemotherapy drugs including paclitaxel and adriamycin in TNBC cells in vitro. It indicates that ADAM10 is associated with chemo-resistance in breast cancer especially in TNBC. But the underlying mechanism is still unknown.

The mechanisms of chemo-resistance in breast cancer include ATP-binding cassette (ABC) transporters such as P-gp transporting a variety of drugs outside the cell membrane, and formation of cancer stem cells (CSCs) which carry CD44 [44]. We previously reported that PrPc promotes multidrug resistance in ER + breast cancer cell line MCF7/ADR by interacting with P-gp [34] and CD44 [26]. On the other hand, ADAM10 proteolytically cleaves and releases many important biologically active substrates including Notch receptors, PrPc [10] and CD44 [45]. In HER2 + breast cancer, ADAM10 cleaves and sheds HER2 fragment p95HER2. Then anti-HER2 antibodies such as trastuzumab cannot bind to p95HER2 which has been proteolytically shed. Therefore, ADAM10 induces HER2 shedding and causes trastuzumab resistance [46]. Based on these investigations, it is hypothesized that ADAM10 may be involved in the drug resistance of TNBC by regulating its substrates associated with chemo-resistance. In TNBC cells, we find that ADAM10 interacts with CD44 and PrPc and initiates their intramembrane proteolysis followed by nuclear transport and signaling of the cytoplasmic domain. These findings confirm our speculation that ADAM10 promotes drug resistance via regulating CD44 and PrPc in TNBC.

In clinical breast cancer patients receiving NACT, we divide them into two cohorts including Cohort 1 with poor response to NACT and Cohort 2 with good response to NACT, based on the effects of NACT. We found that the ADAM10 expression level of pre-NACT tissue samples is significantly higher in Cohort 1 than in Cohort 2. Inversely, after these patients received NACT, ADAM10 protein level of post-NACT samples is distinctly lower in Cohort 1 than in Cohort 2. Also, compared with the pre-NACT samples, the ADAM10 expression level of the post-NACT samples is decreased in Cohort 1 but increased in Cohort 2. We speculate that high ADAM10 protein levels of pre-NACT samples accompanied by overexpression of its substrates, such as PrPc and CD44 in Cohort 1 cases. High substrates levels may impact ADAM10 activity by negative feedback regulation [47]. Consequently, ADAM10 expression is decreased after NACT in Cohort 1 cases. These results suggest that high ADAM10 expression before NACT indicates weak chemotherapy effect. Using an ADAM10 inhibitor in combination with chemotherapeutic drugs may reduce drug resistance and improve therapeutic efficacy. Furthermore, our data showed that high ADAM10 expression is an independent predictor of poor 5-year overall survival in breast cancer patients. However, due to the small number of TNBC cases we collected, the relationship between ADAM10 and the effect of neoadjuvant chemotherapy and overall survival in patients with TNBC could not be determined.

Taking all data together, ADAM10 is involved in the oncogenic process of TNBC and may be provided as a biomarker and therapeutic target in TNBC. In fact, some ADAM10 inhibitors were tested in clinical trials including HER2 + breast cancer [47, 48], but ultimately failed and discontinued due to their ambiguous value. Nevertheless, there is no report on the application of ADAM10 inhibitors in TNBC. In our experiments, ADAM10 affects functions of TNBC by regulating its substrates Notch1 receptor, CD44 and PrPc. Most likely, it will be necessary to first analyze expression of ADAM10 substrates such as Notch receptors, CD44 and PrPc, when selecting patient populations for future ADAM10 inhibitors trials.

## Conclusion

Our study finds that the active form of ADAM10 is highly expressed in TNBC cells, along with the elevated expression of its substrates Notch1, CD44 and PrPc. We have provided evidence that ADAM10 contributes to the pathogenesis of TNBC and promotes drug resistance by activating the Notch signaling pathway and proteolytically regulating CD44 and PrPc. Therefore, ADAM10 may be provided as a helpful biomarker and therapeutic target in TNBC, and the inhibition of ADAM10 could reduce tumor growth, metastasis and chemo-resistance. On the other hand, high ADAM10 expression before NACT predicts unfavorable chemo-therapy response and shorter overall survival.

## Supplementary Information

**Additional file 1: Figure S1.** Effects of ADAM10 down-regulation are detected in ADAM10 knockdown BT-549 cells. a Knockdown efficiency was determined by qRT-PCR after 24h, 48h and 72h transfecting ADAM10 siRNA in BT-549 cells. GAPDH was used as a loading control. b Knockdown efficiency was determined by western blot after 48h and 96h transfecting ADAM10 siRNA in BT-549 cells. GAPDH was used as a loading control. Quantitative analysis of band intensities was conducted in western blot analysis. Knockdown of ADAM10 expression in BT-549 cells attenuated the migration (c) and invasion (d) ability. e CCK8 assay was used for detection of proliferation in BT-549 cells with the use of ADAM10 siRNA or negative control. Bars represent the mean of triplicate samples; error bars represent SD. Data are representative of three independent experiments. The significant difference between BT-549 and BT-549 with negative control or ADAM10 siRNA is indicated by *p < 0.05, **p < 0.01.** Figure S2.** Representative images of transwell chamber assay in MDA-MB-231 cells. Knockdown of ADAM10 expression in MDA-MB-231 cells attenuated the migration (a) and invasion (b) ability. All representative images were taken on power of ×200.

## Data Availability

The data supporting the conclusions of this paper are included within the manuscript.

## References

[1] Seals DF, Courtneidge SA (2003). The ADAMs family of metalloproteases: multidomain proteins with multiple functions. Genes Dev.

[2] Blobel CP (2005). ADAMs: key components in EGFR signalling and development. Nat Rev Mol Cell Biol.

[3] Pruessmeyer J, Ludwig A (2009). The good, the bad and the ugly substrates for ADAM10 and ADAM17 in brain pathology, inflammation and cancer. Semin Cell Dev Biol.

[4] Hartmann D, de Strooper B, Serneels L, Craessaerts K, Herreman A, Annaert W, Umans L, Lubke T, Lena Illert A, von Figura K (2002). The disintegrin/metalloprotease ADAM 10 is essential for Notch signalling but not for alpha-secretase activity in fibroblasts. Hum Mol Genet.

[5] Tsai YH, VanDussen KL, Sawey ET, Wade AW, Kasper C, Rakshit S, Bhatt RG, Stoeck A, Maillard I, Crawford HC (2014). ADAM10 regulates Notch function in intestinal stem cells of mice. Gastroenterology.

[6] Chaimowitz NS, Martin RK, Cichy J, Gibb DR, Patil P, Kang DJ, Farnsworth J, Butcher EC, McCright B, Conrad DH (2011). A disintegrin and metalloproteinase 10 regulates antibody production and maintenance of lymphoid architecture. J Immunol.

[7] Inoshima I, Inoshima N, Wilke GA, Powers ME, Frank KM, Wang Y, Bubeck Wardenburg J (2011). A Staphylococcus aureus pore-forming toxin subverts the activity of ADAM10 to cause lethal infection in mice. Nat Med.

[8] Saftig P, Lichtenthaler SF (2015). The alpha secretase ADAM10: a metalloprotease with multiple functions in the brain. Prog Neurobiol.

[9] Postina R, Schroeder A, Dewachter I, Bohl J, Schmitt U, Kojro E, Prinzen C, Endres K, Hiemke C, Blessing M (2004). A disintegrin-metalloproteinase prevents amyloid plaque formation and hippocampal defects in an Alzheimer disease mouse model. J Clin Investig.

[10] Vincent B, Paitel E, Saftig P, Frobert Y, Hartmann D, De Strooper B, Grassi J, Lopez-Perez E, Checler F (2001). The disintegrins ADAM10 and TACE contribute to the constitutive and phorbol ester-regulated normal cleavage of the cellular prion protein. J Biol Chem.

[11] Altmeppen HC, Prox J, Krasemann S, Puig B, Kruszewski K, Dohler F, Bernreuther C, Hoxha A, Linsenmeier L, Sikorska B (2015). The sheddase ADAM10 is a potent modulator of prion disease. eLife.

[12] Tian L, Wu X, Chi C, Han M, Xu T, Zhuang Y (2008). ADAM10 is essential for proteolytic activation of Notch during thymocyte development. Int Immunol.

[13] Sulis ML, Saftig P, Ferrando AA (2011). Redundancy and specificity of the metalloprotease system mediating oncogenic NOTCH1 activation in T-ALL. Leukemia.

[14] Armanious H, Gelebart P, Anand M, Belch A, Lai R (2011). Constitutive activation of metalloproteinase ADAM10 in mantle cell lymphoma promotes cell growth and activates the TNFalpha/NFkappaB pathway. Blood.

[15] Gavert N, Sheffer M, Raveh S, Spaderna S, Shtutman M, Brabletz T, Barany F, Paty P, Notterman D, Domany E (2007). Expression of L1-CAM and ADAM10 in human colon cancer cells induces metastasis. Can Res.

[16] Knosel T, Emde A, Schluns K, Chen Y, Jurchott K, Krause M, Dietel M, Petersen I (2005). Immunoprofiles of 11 biomarkers using tissue microarrays identify prognostic subgroups in colorectal cancer. Neoplasia.

[17] Ko SY, Lin SC, Wong YK, Liu CJ, Chang KW, Liu TY (2007). Increase of disintergin metalloprotease 10 (ADAM10) expression in oral squamous cell carcinoma. Cancer Lett.

[18] Gaida MM, Haag N, Gunther F, Tschaharganeh DF, Schirmacher P, Friess H, Giese NA, Schmidt J, Wente MN (2010). Expression of A disintegrin and metalloprotease 10 in pancreatic carcinoma. Int J Mol Med.

[19] Kohutek ZA, diPierro CG, Redpath GT, Hussaini IM (2009). ADAM-10-mediated N-cadherin cleavage is protein kinase C-alpha dependent and promotes glioblastoma cell migration. J Neurosci.

[20] Liu PC, Liu X, Li Y, Covington M, Wynn R, Huber R, Hillman M, Yang G, Ellis D, Marando C (2006). Identification of ADAM10 as a major source of HER2 ectodomain sheddase activity in HER2 overexpressing breast cancer cells. Cancer Biol Ther.

[21] Feldinger K, Generali D, Kramer-Marek G, Gijsen M, Ng TB, Wong JH, Strina C, Cappelletti M, Andreis D, Li JL (2014). ADAM10 mediates trastuzumab resistance and is correlated with survival in HER2 positive breast cancer. Oncotarget.

[22] Zheng H, Zhong A, Xie S, Wang Y, Sun J, Zhang J, Tong Y, Chen M, Zhang G, Ma Q (2019). Elevated serum HER-2 predicts poor prognosis in breast cancer and is correlated to ADAM10 expression. Cancer Med.

[23] Lee JY, Joo HS, Choi HJ, Jin S, Kim HY, Jeong GY, An HW, Park MK, Lee SE, Kim WS (2018). Role of MEL-18 amplification in anti-HER2 therapy of breast cancer. J Natl Cancer Inst.

[24] Garrido-Castro AC, Lin NU, Polyak K (2019). Insights into molecular classifications of triple-negative breast cancer: improving patient selection for treatment. Cancer Discov.

[25] Mullooly M, McGowan PM, Kennedy SA, Madden SF, Crown J, O’Donovan N, Duffy MJ (2015). ADAM10: a new player in breast cancer progression?. Br J Cancer.

[26] Cheng Y, Tao L, Xu J, Li Q, Yu J, Jin Y, Chen Q, Xu Z, Zou Q, Liu X (2014). CD44/cellular prion protein interact in multidrug resistant breast cancer cells and correlate with responses to neoadjuvant chemotherapy in breast cancer patients. Mol Carcinog.

[27] Ogston KN, Miller ID, Payne S, Hutcheon AW, Sarkar TK, Smith I, Schofield A, Heys SD (2003). A new histological grading system to assess response of breast cancers to primary chemotherapy: prognostic significance and survival. Breast.

[28] Goulding H, Pinder S, Cannon P, Pearson D, Nicholson R, Snead D, Bell J, Elston CW, Robertson JF, Blamey RW (1995). A new immunohistochemical antibody for the assessment of estrogen receptor status on routine formalin-fixed tissue samples. Hum Pathol.

[29] Locatelli M, Curigliano G (2017). Notch inhibitors and their role in the treatment of triple negative breast cancer: promises and failures. Curr Opin Oncol.

[30] Qiu M, Peng Q, Jiang I, Carroll C, Han G, Rymer I, Lippincott J, Zachwieja J, Gajiwala K, Kraynov E (2013). Specific inhibition of Notch1 signaling enhances the antitumor efficacy of chemotherapy in triple negative breast cancer through reduction of cancer stem cells. Cancer Lett.

[31] Weber S, Niessen MT, Prox J, Lullmann-Rauch R, Schmitz A, Schwanbeck R, Blobel CP, Jorissen E, de Strooper B, Niessen CM (2011). The disintegrin/metalloproteinase Adam10 is essential for epidermal integrity and Notch-mediated signaling. Development.

[32] Hartmann M, Parra LM, Ruschel A, Lindner C, Morrison H, Herrlich A, Herrlich P (2015). Inside-out regulation of ectodomain Cleavage of Cluster-of-Differentiation-44 (CD44) and of neuregulin-1 requires substrate dimerization. The Journal of biological chemistry.

[33] Idowu MO, Kmieciak M, Dumur C, Burton RS, Grimes MM, Powers CN, Manjili MH (2012). CD44(+)/CD24(−/low) cancer stem/progenitor cells are more abundant in triple-negative invasive breast carcinoma phenotype and are associated with poor outcome. Hum Pathol.

[34] Li QQ, Cao XX, Xu JD, Chen Q, Wang WJ, Tang F, Chen ZQ, Liu XP, Xu ZD (2009). The role of P-glycoprotein/cellular prion protein interaction in multidrug-resistant breast cancer cells treated with paclitaxel. Cell Mol Life Sci.

[35] Cancer Genome Atlas N (2012). Comprehensive molecular portraits of human breast tumours. Nature.

[36] Wetzel S, Seipold L, Saftig P (2017). The metalloproteinase ADAM10: a useful therapeutic target?. Mol Cell Res.

[37] Tsang JYS, Lee MA, Chan TH, Li J, Ni YB, Shao Y, Chan SK, Cheungc SY, Lau KF, Tse GMK (2018). Proteolytic cleavage of amyloid precursor protein by ADAM10 mediates proliferation and migration in breast cancer. EBioMedicine.

[38] Krishna BM, Jana S, Singhal J, Horne D, Awasthi S, Salgia R, Singhal SS (2019). Notch signaling in breast cancer: From pathway analysis to therapy. Cancer Lett.

[39] Reedijk M, Odorcic S, Chang L, Zhang H, Miller N, McCready DR, Lockwood G, Egan SE (2005). High-level coexpression of JAG1 and NOTCH1 is observed in human breast cancer and is associated with poor overall survival. Can Res.

[40] Borggrefe T, Oswald F (2009). The Notch signaling pathway: transcriptional regulation at Notch target genes. Cell Mol Life Sci.

[41] Ding ZY, Li R, Zhang QJ, Wang Y, Jiang Y, Meng QY, Xi QL, Wu GH (2019). Prognostic role of cyclin D2/D3 in multiple human malignant neoplasms: a systematic review and meta-analysis. Cancer Med.

[42] Niimi H, Pardali K, Vanlandewijck M, Heldin CH, Moustakas A (2007). Notch signaling is necessary for epithelial growth arrest by TGF-beta. J Cell Biol.

[43] Takebe N, Harris PJ, Warren RQ, Ivy SP (2011). Targeting cancer stem cells by inhibiting Wnt, Notch, and Hedgehog pathways. Nat Rev Clin Oncol.

[44] Ji X, Lu Y, Tian H, Meng X, Wei M, Cho WC (2019). Chemoresistance mechanisms of breast cancer and their countermeasures. Biomed Pharmacother.

[45] Parra LM, Hartmann M, Schubach S, Li Y, Herrlich P, Herrlich A (2015). Distinct intracellular domain substrate modifications selectively regulate ectodomain cleavage of NRG1 or CD44. Mol Cell Biol.

[46] Scaltriti M, Nuciforo P, Bradbury I, Sperinde J, Agbor-Tarh D, Campbell C, Chenna A, Winslow J, Serra V, Parra JL (2015). High HER2 expression correlates with response to the combination of lapatinib and trastuzumab. Clin Cancer Res.

[47] Miller MA, Oudin MJ, Sullivan RJ, Wang SJ, Meyer AS, Im H, Frederick DT, Tadros J, Griffith LG, Lee H (2016). Reduced proteolytic shedding of receptor tyrosine kinases is a post-translational mechanism of kinase inhibitor resistance. Cancer Discov.

[48] Witters L, Scherle P, Friedman S, Fridman J, Caulder E, Newton R, Lipton A (2008). Synergistic inhibition with a dual epidermal growth factor receptor/HER-2/neu tyrosine kinase inhibitor and a disintegrin and metalloprotease inhibitor. Can Res.

