# Supplementary material for: ADAM10 is involved in the oncogenic process and chemo-resistance of triple-negative breast cancer via regulating Notch1 signaling pathway, CD44 and PrPc
Source: Cancer Cell Int. 2021 Jan 7;21:32. doi: 10.1186/s12935-020-01727-5 (PMC7791678; doi:10.1186/s12935-020-01727-5)
Supplement: Supplementary file 1 — Additional file 1: Figure S1. Effects of ADAM10 down-regulation are detected in ADAM10 knockdown BT-549 cells. a Knockdown efficiency was determined by qRT-PCR after 24h, 48h and 72h transfecting ADAM10 siRNA in BT-549 cells. GAPDH was used as a loading control. b Knockdown efficiency was determined by western blot after 48h and 96h transfecting ADAM10 siRNA in BT-549 cells. GAPDH was used as a loading control. Quantitative analysis of band intensities was conducted in western blot analysis. Knockdown of ADAM10 expression in BT-549 cells attenuated the migration (c) and invasion (d) ability. e CCK8 assay was used for detection of proliferation in BT-549 cells with the use of ADAM10 siRNA or negative control. Bars represent the mean of triplicate samples; error bars represent SD. Data are representative of three independent experiments. The significant difference between BT-549 and BT-549 with negative control or ADAM10 siRNA is indicated by *p < 0.05, **p < 0.01. Figure S2. Representative images of transwell chamber assay in MDA-MB-231 cells. Knockdown of ADAM10 expression in MDA-MB-231 cells attenuated the migration (a) and invasion (b) ability. All representative images were taken on power of ×200. [file 12935_2020_1727_MOESM1_ESM.doc]

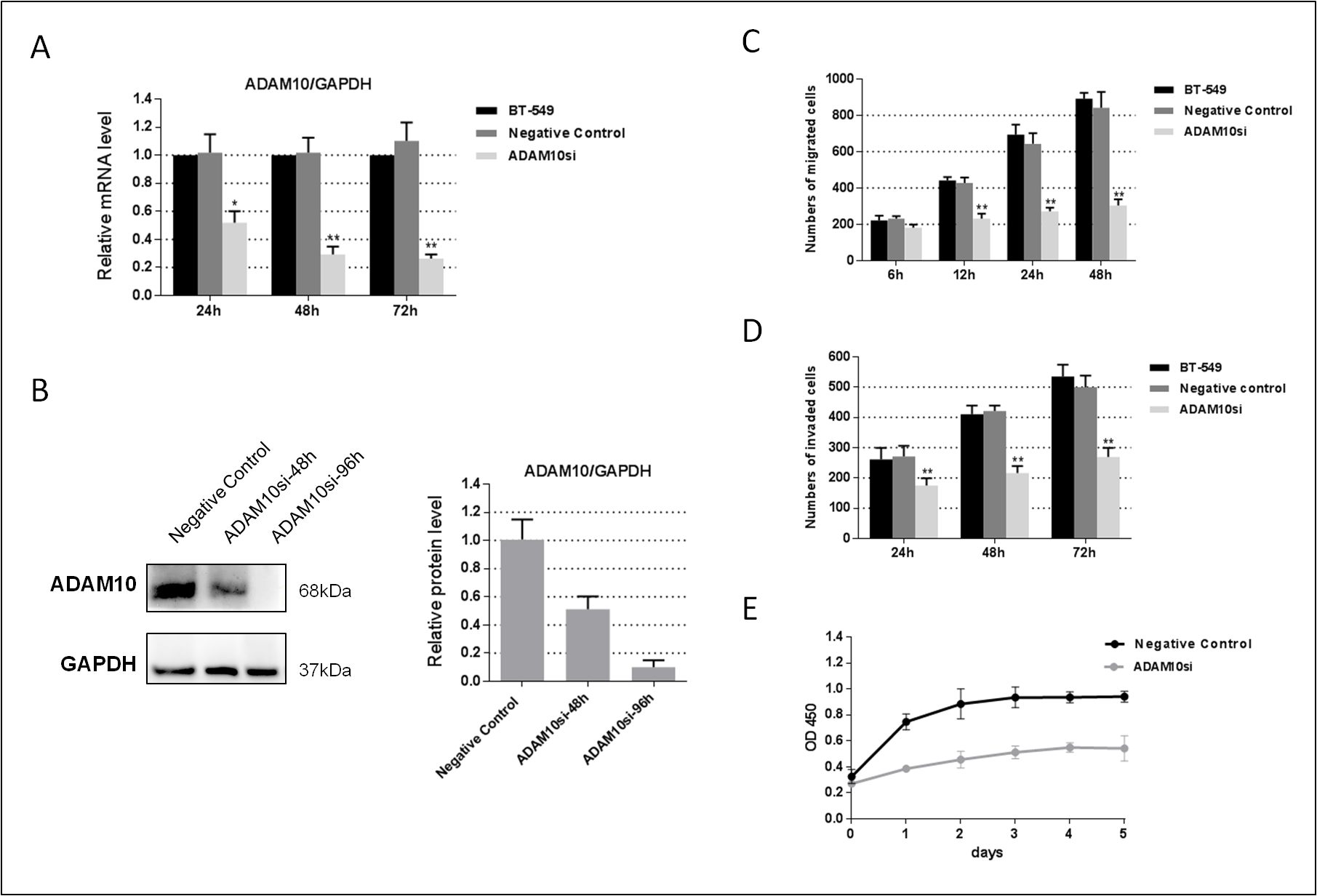


**Fig. S1 Effects of ADAM10 down-regulation are detected in ADAM10 knockdown BT-549 cells.**

**a** Knockdown efficiency was determined by qRT-PCR after 24h, 48h and 72h transfecting ADAM10 siRNA in BT-549 cells. GAPDH was used as a loading control. **b** Knockdown efficiency was determined by western blot after 48h and 96h transfecting ADAM10 siRNA in BT-549 cells. GAPDH was used as a loading control. Quantitative analysis of band intensities was conducted in western blot analysis. Knockdown of ADAM10 expression in BT-549 cells attenuated the migration (**c**) and invasion (**d**) ability. **e** CCK8 assay was used for detection of proliferation in BT-549 cells with the use of ADAM10 siRNA or negative control. Bars represent the mean of triplicate samples; error bars represent SD. Data are representative of three independent experiments. The significant difference between BT-549 and BT-549 with negative control or ADAM10 siRNA is indicated by **p* < 0.05, ***p* < 0.01.


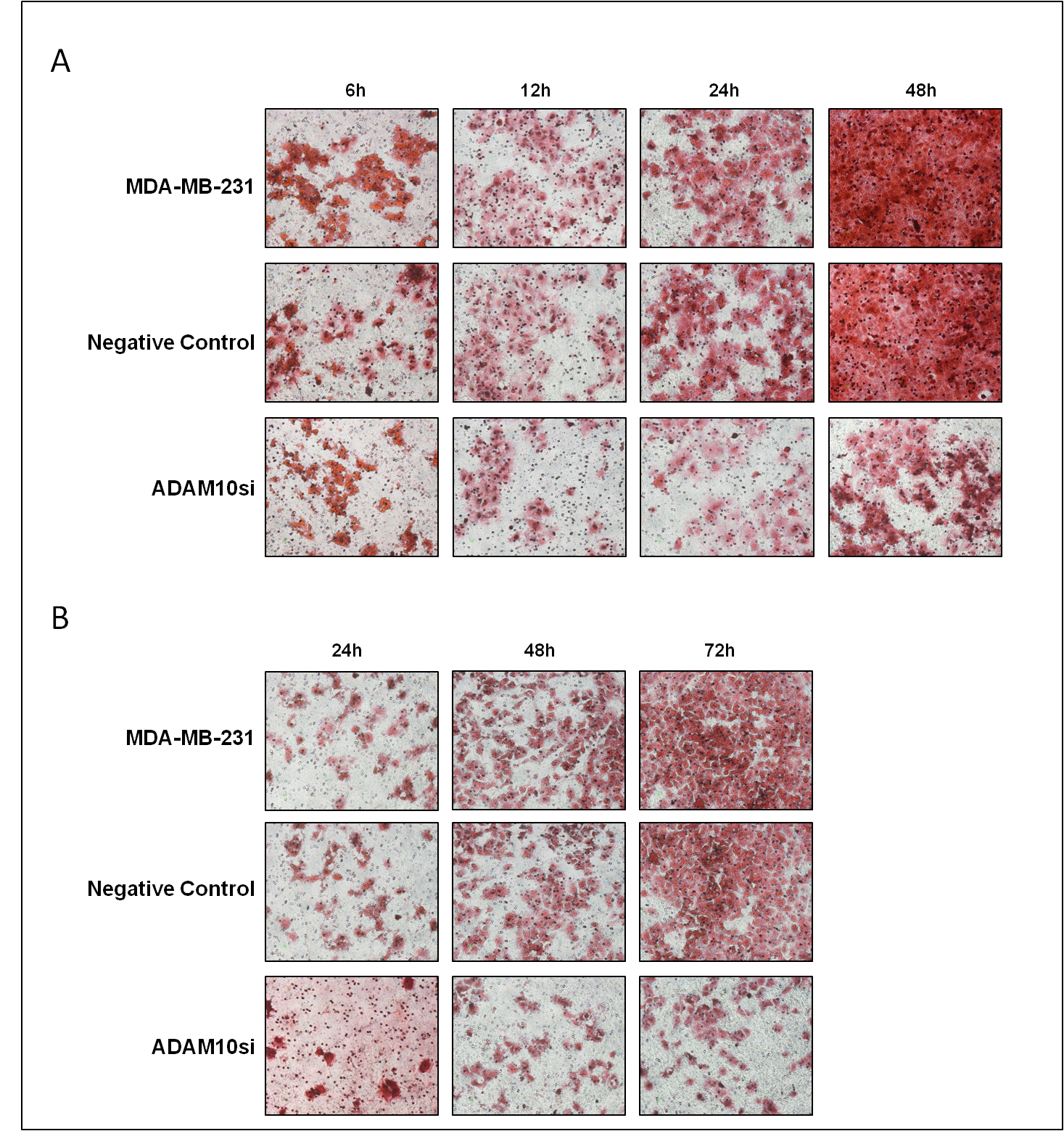


**Fig. S2 Representative images of transwell chamber assay in MDA-MB-231 cells.**

Knockdown of ADAM10 expression in MDA-MB-231 cells attenuated the migration (**a**) and invasion (**b**) ability. All representative images were taken on power of ×200.
